# On some problems of Bayesian region construction with guaranteed coverages

**DOI:** 10.1007/s00362-023-01394-4

**Published:** 2023-01-24

**Authors:** Michael Evans, Miaoshiqi Liu, Michael Moon, Sabrina Sixta, Siyi Wei, Siyue Yang

**Affiliations:** Department of Statistical Sciences, University of Toronto, 700 University Ave, Toronto, ON M5G 1Z5 Canada

**Keywords:** Relative belief, Plausible region, Bias against, Bias in favor, Coverage, Accuracy

## Abstract

The general problem of constructing regions that have a guaranteed coverage probability for an arbitrary parameter of interest $\psi \in \Psi$ is considered. The regions developed are Bayesian in nature and the coverage probabilities can be considered as Bayesian confidences with respect to the model obtained by integrating out the nuisance parameters using the conditional prior given $\psi .$ Both the prior coverage probability and the prior probability of covering a false value (the accuracy) can be controlled by setting the sample size. These coverage probabilities are considered as a priori figures of merit concerning the reliability of a study while the inferences quoted are Bayesian. Several problems are considered where obtaining confidence regions with desirable properties have proven difficult to obtain. For example, it is shown that the approach discussed never leads to improper regions which has proven to be an issue for some confidence regions.

## Introduction

The confidence concept arises in statistics as follows: there is a statistical model $\{f_{\theta }:\theta \in \Theta \}$ for data $x\in {\mathcal {X}},$ a marginal parameter of interest $\psi =\Psi (\theta ),$ where $\Psi :\Theta \overset{onto}{\rightarrow }\Psi$ (with the same notation used here for the function and its range), a desired confidence level $\gamma \in (0,1)$ and the goal is to state a region $C(x)\subset \Psi$ such that $P_{\theta }(\Psi (\theta )\in C(x))\ge \gamma$ for every $\theta \in \Theta .$ While there can be different motivations for reporting such a region, the one considered here is that there is an estimate $\psi (x)$ of the parameter of interest such that $\psi (x)\in C(x)$ and the “size” of *C*(*x*),  together with the confidence $\gamma ,$ serve as an assessment of the accuracy of the recorded estimate. It is well-known that confidence regions can sometimes give improper answers as discussed, for example, in Plante (2020). By improper here is meant that *C*(*x*) could be the null set or all of $\Psi$ with positive probability,  and so be uninformative. In such situations it is difficult to see how reporting *C*(*x*) can be regarded as a valid assessment of the accuracy of $\psi (x).$

The problem of error assessment via quoting a region *C*(*x*), can also be approached by adding a prior $\pi$ to the problem and providing a Bayesian credible region having posterior content at least $\gamma .$ The Bayesian approach has the virtues of the error assessment being based on the observed data and such a region can always be constructed, say via the hpd (highest posterior density) principle. There are criticisms that can be leveled at this approach, however, as there is no assessment of the reliability of the inference which is implicit in the frequentist approach via repeated sampling. While the use of a prior is also sometimes criticized, the position taken here is that this is no different than the use of a statistical model as, while the model can be checked for its agreement with the observed data via model checking, similarly a prior can be submitted to a check for prior-data conflict, see Evans and Moshonov (2006), Evans (2015) and Nott et al. (2020). There is also the issue of bias which is interpreted here as meaning that the ingredients to the analysis, namely, the data collection procedure together with the model and prior, can be chosen in such a fashion as to produce a foregone conclusion. That such bias is possible is illustrated in Evans (2015) and Evans and Guo (2021) where also a solution to this issue is developed.

Rather than invoke something like the hpd principle to construct a credible region, the approach taken here is somewhat different. This is based on the *principle of evidence*: there is evidence in favor of a value $\psi$ if its posterior probability has increased over its prior probability, evidence against $\psi$ if the posterior probability has decreased and there is no evidence either way if they are equal. This simple principle has broad implications not the least of which being that it makes little sense to allow any reported region to include a value for which there is evidence against it being true. In fact, a reported credible region can contain $\psi$ values for which there is evidence against $\psi$ being the true value. As such, it is more appropriate to quote what is called here the * plausible region *$Pl_{\Psi }(x)$*,* namely, those values of $\psi$ for which there is evidence in favor of $\psi$ being true, see Evans (2015) and Sect. 2. The principle of evidence also leads to a direct method for measuring and controlling bias which comes in two forms for this problem. Here the *implausible region* $Im_\Psi (x)$, refers to the set of ${\psi }$ values for which evidence against is obtained.

(i) *Bias against* refers to the prior probability that $Pl_{\Psi }(x)$ does not contain the true value.
(ii) *Bias in favor* refers to the prior probability that $\ Im_\Psi (x)$ does not contain a meaningfully false value as defined in Sect. 2.

As discussed in Evans and Guo (2021), the control of bias is equivalent to the a priori control of coverage probabilities. The control over the biases is effected by ensuring that an appropriate amount of data is collected as it can be shown that both biases converge to 0 as the amount of data increases. So the biases are controlled at the planning stage of a statistical investigation. Controlling bias against is equivalent to controlling the prior probability of $Pl_{\Psi }(x)$ containing the true value. This coverage probability will be referred to here as the *Bayesian confidence* level of the plausible region as the coverage probability is with respect to the marginal model obtained by integrating out the nuisance parameters using the conditional prior given the parameter of interest. This approach can also give a pure confidence, namely, with respect to the original model, when the parameter of interest is the full model parameter $\theta =\Psi (\theta ).$ Controlling bias in favor is typically equivalent to setting the *accuracy* of $Pl_{\Psi }(x)$ where accuracy refers to the probability of a region covering false values. This is better expressed through the prior probability of $\ Im_{\Psi }(x)$ containing a meaningfully false value of $\psi$ where “meaningfully false” is defined in Sect. 2. Again the accuracy is with respect to the marginal model obtained by integrating out nuisance parameters. The definitions and results concerning the biases are more fully discussed in Sect. 2.

The end result of this approach is the best of both approaches to the problem, namely, a Bayesian region with a particular posterior content that reflects the uncertainty in the observed data, together with a guaranteed Bayesian confidence and accuracy, that reflects the reliability of the inference. The reliability of an inference refers to the extent to which an inference is trustworthy and, in general, Bayesian inferences do not address this issue. It is important to note that these results hold for any proper prior and, at least up to computational difficulties, can always be implemented. In particular, there is no need to search for a prior that will provide an appropriate Bayesian confidence. So, an elicited prior can be used, and there is no need for the posterior content and the Bayesian confidence to agree, as they refer to different aspects of a statistical investigation. The final inference is an estimate $\psi (x)$ of $\psi ,$ the plausible region $Pl_{\Psi }(x)$ and its posterior content,  where $\psi (x)\in Pl_{\Psi }(x)$ and a measure of the “size” of $Pl_{\Psi }(x).$ The posterior content of $Pl_{\Psi }(x)$ reflects how strongly it is believed that the true value is in this set while the size measures how accurate the estimate is. The measurement of size is context dependent and might simply be the Euclidean volume of $Pl_{\Psi }(x)$ when this makes sense. The overall point to be noted about the approach taken here is that inferences are Bayesian but frequentism plays a key role in the design of the study through the control of the biases.

Section 2 discusses some necessary background and establishes the new result that a plausible region is never improper. Section 3 applies this approach to several well-known problems where the construction of frequentist confidence regions has proven to be at the very least difficult and, one could argue, for which there is no current satisfactory solution. The methodology is general and can be applied to any problem with a Bayesian formulation using proper priors and so this provides a degree of unification between Bayes and frequentism.

It is to be noted that the problem considered can be characterized as one of scientific inference. In this context there is no preferred utility or loss function employed but rather a summary is required of what the evidence in the data has to say about the quantity of interest $\psi .$ It may be that a decision based on a loss or utility is relevant in a problem but even then it is of interest to see what the evidence says, especially if this results in a contradiction. For example, the evidence from a clinical trial may indicate that a vaccine is efficacious in preventing a disease but a decision is made to not market the vaccine due to side effects, costs, etc.

## Relative belief inferences and bias

If the prior and posterior densities of $\psi$ are denoted by $\pi _{\Psi }$ and $\pi _{\Psi }(\cdot \,\vert \,x),$ then the relative belief ratio of $\psi$ is given by $RB_{\Psi }(\psi \,\vert \,x)=\pi _{\Psi }(\psi \,\vert \,x)/\pi _{ \Psi }(\psi ).$ There is then evidence in favor of $\psi$ when $RB_{\Psi }(\psi \,\vert \,x)>1,$ evidence against when $RB_{\Psi }(\psi \,\vert \,x)<1$ and no evidence either way when $RB_{\Psi }(\psi \,\vert \,x)=1.$ This follows from the principle of evidence when the prior distribution of $\psi$ is discrete and the following limiting argument in the general case. If $A_{\varepsilon }(\psi )$ is a sequence of sets converging nicely (see Rudin (1974) for the definition) to $\{\psi \}$ as $\varepsilon \rightarrow 0,$ then, with $\Pi _{\Psi }$ and $\Pi _{\Psi }(\cdot \,\vert \,x)$ denoting the prior and posterior measures of $\psi ,$

$$\begin{aligned} \lim _{\varepsilon \rightarrow 0}RB(A_{\varepsilon }(\psi )\,\vert \,x)=\lim _{ \varepsilon \rightarrow 0}\frac{\Pi _{\Psi }(A_{\varepsilon }(\psi )\,\vert \,x)}{ \Pi _{\Psi }(A_{\varepsilon }(\psi ))}=\frac{\pi _{\Psi }(\psi \,\vert \,x)}{ \pi _{\Psi }(\psi )} \end{aligned}$$

whenever the prior $\pi _{\Psi }$ is positive and continuous at $\psi .$ For example, taking $A_{\varepsilon }(\psi )$ to be the ball centered at $\psi$ of radius $\varepsilon$ gives this result but other choices are possible. Actually, for much of the discussion here, any valid measure of evidence can be used instead of the relative belief ratio, where *valid* means there is a cut-off that determines evidence against versus evidence in favor according to the principle of evidence. For example, a Bayes factor is a valid measure of evidence also using the value 1 as the cut-off. As will be seen, the plausible region and the measures of bias are independent of the valid measure of evidence used so this is not an issue for the discussion here.

The set of values for which there is evidence in favor is the plausible region $Pl_{\Psi }(x)=\{\psi :RB_{\Psi }(\psi \,\vert \,x)>1\}.$ When the $\psi$ values are ordered by the amount of evidence using the relative belief ratio, the natural estimate of $\psi$ is given by $\psi (x)=\arg \sup _{ \psi }RB_{\Psi }(\psi \,\vert \,x)\in Pl_{\Psi }(x).$ The posterior content of $Pl_{\Psi }(x)$ measures how strongly it is believed that the true value is in $Pl_{\Psi }(x).$ When $\Psi$ is an open subset of a Euclidean space it typically makes sense to report the volume of $Pl_{\Psi }(x)$ as a measure of its size. Volume doesn’t work, however, in the non-Euclidean case, for example $\psi$ is a graph, and so some other measure of size is required. The choice of the size measure is context dependent and could simply be counting measure when $\psi$ is a discrete parameter, but the prior probability content of $Pl_{\Psi }(x)$ also gives a general measure of size. So $\psi (x)$ can be considered a highly accurate estimate when the posterior probability $\Pi _{\Psi }(Pl_{\Psi }(x)\,\vert \,x)$ is high, as then there is a high degree of belief the true value is in $Pl_{\Psi }(x),$ and the prior probability $\Pi _{\Psi }(Pl_{\Psi }(x))$ is small. Note that the prior reflects initial beliefs about the true value of $\psi$ so such an outcome indicates that a great deal has been learned relative to the prior. Also, any other estimate determined in this way from a valid measure of evidence will also lie in $Pl_{\Psi }(x)$ and so produces no gain in accuracy over $\psi (x).$

It is possible, however, that there is bias in Bayesian inferences. For example, suppose that the goal is to assess the hypothesis $H_{0}:\Psi (\theta )=\psi _{0}.$ The relative belief ratio $RB_{\Psi }(\psi _{0}\,\vert \,x)$ indicates whether there is evidence in favor of or against $H_{0}$ and there are several approaches to measuring the strength of this evidence but this is not considered further here, see Evans (2015). Suppose that evidence against $H_{0}$ is obtained but that there is a large prior probability of not getting evidence in favor even when $H_{0}$ is true, namely, the probability

1 $$\begin{aligned} \text {bias against}_{\Psi }(\psi _{0})=M(RB_{\Psi }(\psi _{0}\,\vert \,x)\le 1\, \vert \psi _{0}) \end{aligned}$$

is large where $$M(\cdot \,\vert \,\psi _{0})$$ denotes the conditional prior measure of the data given that $$H_{0}$$ is true, namely, $$M(A\,\vert \,\psi _{0})=\int _A \int _{\textcircled {H}} f_\theta (x) \, \Pi (d\theta \,\vert \,\psi _{0})dx$$ and $$\Pi (\cdot \,\vert \,\psi _{0})$$ is the conditional prior measure for $$\theta $$ given that $$\Psi (\theta )=\psi _0$$. So $$M(\cdot \,\vert \,\psi _{0})$$ is obtained by integrating out the nuisance parameters. It seems reasonable then to treat the finding of evidence against $$H_{0}$$ as unreliable and it can be said that there is an a priori *bias against* $$H_{0}.$$ Similarly, using a metric *d* on $$\Psi ,$$ if evidence in favor of $$ H_{0}$$ is obtained but

2 $$\begin{aligned} \text {bias in favor}_{\Psi }(\psi _{0})=\sup _{\psi :d(\psi _{0},\psi )\ge \delta }M(RB_{\Psi }(\psi _{0}\,\vert \,x)\ge 1\,\vert \,\psi ) \end{aligned}$$

is large, namely, there is a large prior probability of not obtaining evidence against $$H_{0}$$ when it is *meaningfully false*, namely, $$\int _A \int _{\textcircled {H}} f_\theta (x) \, \Pi (d\theta \,\vert \,\psi _{0})dx$$ where the specification of $$\delta $$ is discussed in the following paragraph,  then it is said that there is *bias in favor* of $$H_{0}.$$ Note that $$M(RB_{\Psi }(\psi _{0}\,\vert \,x)\ge 1\,\vert \,\psi )$$ generally decreases as $$\psi $$ moves away from $$ \psi _{0},$$ so it is then only necessary to consider values of $$\psi $$ satisfying $$d(\psi _{0},\psi )=\delta $$ to determine the bias in favor. Clearly there is some similarity between the frequentist size and power of a test and the bias against and bias in favor here but there is no suggestion that we are to accept or reject $$H_{0}.$$ The purpose of the biases is to measure the reliability of what the evidence in the observed data tells us about $$ H_{0}.$$

The value of $\delta$ is not arbitrary but is determined by the application, as it represents the accuracy to which it is desired to know the true value of $\psi$ and is thus part of the design. For example, if interest is in inference about the mean of a response variable that is measured to the nearest centimeter in the data collected, then the true value of the mean can only be known to the nearest centimeter, no matter how large the sample size is, and the measurement process then determines that $\delta \ge 0.5$ cm. If greater accuracy is desired then a more precise measurement process must be employed. The value of $\delta$ can be considered as expressing what is meant by scientific significance as opposed to statistical significance and the need for this distinction has long been recognized, see Boring (1919). Although the specification of $\delta$ isn’t always made, such usage does arise in studies where sample size is chosen to make the power of a test such that a meaningful deviation from a null hypothesis can be detected with high probability. The point-of-view taken here is that specifying such a $\delta$ is part of good statistical practice. If, for a variety of reasons $\delta$ is not available, then the analysis can be carried out for several values to assess the sensitivity of the conclusions to such a choice.

The probability measures $M(\cdot \,\vert \,\psi )$ depend on the prior $\pi$ only through the conditional prior $$\pi (\cdot \,\vert \,\psi )\ $$and do not depend on the marginal prior $\pi _{\Psi }$ for the parameter of interest. As such the probabilities determined by $M(\cdot \,\vert \,\psi )$ are essentially frequentist in nature and similar to the use of distributions on parameters in mixed models, namely, $\pi (\cdot \,\vert \,\psi )$ is used to integrate out nuisance parameters. The bias probabilities (1) and (2) are exactly frequentist but for the model given by $\{m(\cdot \,\vert \,\psi ):\psi \in \Psi \},$ where $m(\cdot \,\vert \,\psi )$ is the density of $M(\cdot \,\vert \,\psi ),$ and this corresponds to the original model when $\Psi (\theta )=\theta .$

The average bias against a value of $\psi \sim \pi _{\Psi }$ can be written as

3 $$\begin{aligned} \text {bias against}_{\Psi }&=E_{\Pi _{\Psi }}(M(RB_{\Psi }(\psi \,\vert \,x)\le 1\,\vert \,\psi ))=E_{\Pi _{\Psi }}(M(\psi \notin Pl_{\Psi }(x)\,\vert \,\psi )) \nonumber \\&=1-E_{\Pi _{\Psi }}(M(\psi \in Pl_{\Psi }(x)\,\vert \,\psi )). \end{aligned}$$

So (3) is determined by the prior coverage probability $E_{\Pi _{\Psi }}(M(\psi \in Pl_{\Psi }(x)\,\vert \,\psi ))$ of the plausible region which will be referred to here as a Bayesian confidence as it is the prior probability that $Pl_{\Psi }(x)$ will contain the true value. Note that, typically there is an upper bound for $M(RB_{\Psi }(\psi \,\vert \,x)\le 1\,\vert \,\psi )$ as a function of $\psi ,$ so 1 minus this bound serves as a lower bound on the Bayesian confidence. This lower bound is then a frequentist confidence but with respect to the model $\{m(\cdot \,\vert \,\psi ):\psi \in \Psi \}$ which gives a lower bound on the pure frequentist confidence when $\Psi (\theta )=\theta$. Appendix A contains some pseudocode that outlines a general computational procedures that can be used to compute these quantities.

Also the average bias in favor can be written as

4 $$\begin{aligned} \text {bias in favor}_{\Psi }&=E_{\Pi _{\Psi }}\left( \sup _{\psi _{*}:d(\psi ,\psi _{*})\ge \delta }M(RB_{\Psi }(\psi \,\vert  x)\ge 1\,\vert \,\psi _{*})\right) \nonumber \\&=E_{\Pi _{\Psi }}\left( \sup _{\psi _{*}:d(\psi ,\psi _{*})\ge \delta }M(\psi \notin \ Im_\Psi (x)\,\vert \,\psi _{*})\right) , \end{aligned}$$

which is the prior probability that a meaningfully false value is not in the *implausible region* $\ Im_\Psi (x)=\{\psi :RB_{\Psi }(\psi \, \vert \,x)<1\},$ the set of values for which there is evidence against. In cases where the prior distribution of $\psi$ is continuous, then typically ( 4) is an upper bound on the prior probability of $Pl_{\Psi }(x)$ covering a meaningfully false value.

While it might be appealing to consider choosing the prior to make both these biases small, this is the wrong approach as indeed experience indicates that choosing a prior to minimize bias against simply increases bias in favor and conversely. As discussed in Evans and Guo (2021), as the diffuseness of the prior increases, typically bias in favor increases and bias against decreases. The way to control these biases is, as established in Evans (2015), through the amount of data collected as both biases converge to 0 as this increases. As such, it is possible to control both the prior probability of $Pl_{\Psi }(x)$ covering the true value and the prior probability of it covering a meaningfully false value and so obtain a Bayesian inference with good frequentist properties. Of course, this is similar to the use of coverage probabilities in frequentist inference but the reported inferences are indeed Bayesian while the biases are concerned with ensuring that the inferences are reliable from a frequentist perspective.

A region *C* for $\Psi$ is called *improper* if it is possible that $C(x)=\phi$ or $C(x)=\Psi$ with positive probability. Theorem 1 establishes that plausible regions can never be improper in realistic statistical contexts. The result can be viewed as a logical consistency result for this approach to assessing the error in an estimate. For this let $m(x)=\int _{\Theta }f_{\theta }(x)\,\Pi (d\theta )$ denote the prior predictive density associated with the corresponding measure $M,m(x\,\vert \,\psi )= \int _{\Theta }f_{\theta }(x)\,\Pi (d\theta \,\vert \,\psi )$ be the conditional prior predictive density of the data given $\Psi (\theta )=\psi$ and put

$$\begin{aligned} F=\{x:RB_{\Psi }(\psi \,\vert \,x)=1\text { a.e. }\Pi _{\Psi }\}=\{x:m(x\,\vert \, \psi )=m(x)\text { a.e. }\Pi _{\Psi }\} \end{aligned}$$

where a.e. $\Pi _{\Psi }$ means the condition holds with $\Pi _{\Psi }$ probability 1. The last equality follows from the Savage-Dickey ratio result, namely, using $J_{\Psi }(\theta )=\vert \det (d\Psi (\theta )(d\Psi (\theta ))^{t})\vert ^{-1/2},$ where $d\Psi (\theta )$ is the Jacobian matrix of the transformation $\Psi ,$ then

$$\begin{aligned}&RB_{\Psi }(\psi \,\vert \,x)=\frac{\pi _{\Psi }(\psi \,\vert \,x)}{ \pi _{\Psi }(\psi )}=\frac{\int _{\Psi ^{-1}\{\psi \}}\pi (\theta \,\vert \,x)J_{ \Psi }(\theta )\,d\theta }{\pi _{\Psi }(\psi )} \\&=\int _{\Psi ^{-1}\{\psi \}}\frac{f_{\theta }(x)}{m(x)}\frac{\pi (\theta )J_{\Psi }(\theta )}{\pi _{\Psi }(\psi )}\,d\theta =\int _{\Psi ^{-1}\{\psi \}}\frac{ f_{\theta }(x)\pi (\theta \,\vert \,\psi )}{m(x)}\,d\theta =\frac{ m(x\,\vert \,\psi )}{m(x)} \end{aligned}$$

where the integration is with respect to volume on $\Psi ^{-1}\{\psi \}$ and the conditional prior density of $\theta$ given $\Psi (\theta )=\psi$ equals $\pi (\theta \,\vert \,\psi )=\pi (\theta )J_{\Psi }(\theta )/\pi _{\Psi }(\psi ).$ See Appendix A of Evans (2015) for the smoothness conditions required for the formulas used here for $\pi _{\Psi }(\psi \,\vert \,x)$ and $\pi (\theta \,\vert \,\psi ).$ In particular, in the discrete case the integral is a sum and $J_{\Psi }(\theta )\equiv 1.$ Note that, the conditional prior distribution of the data given *F* has no dependence on the parameter of interest and, except in extraordinary circumstances, this set will have prior probability 0, namely, $M(F)=0.$ For, if $x\in F,$ then nothing can be learned as there is no evidence in either direction for any value of $\psi .$

### Theorem 1

The plausible region for $\psi =\Psi (\theta )$ (i) never satisfies $Pl_{\Psi }(x)=\Psi$ and (ii) satisfies $Pl_{\Psi }(x)=\phi$ with prior probability 0 when $M(F)=0.$

### Proof

(i) Suppose that $Pl_{\Psi }(x)=\Psi .$ This is true iff $RB_{\Psi }(\psi \,\vert \,x)>1$ for every $$\psi \ $$and so

$$\begin{aligned} 1<\int _{\Psi }RB_{\Psi }(\psi \,\vert \,x)\,\Pi _{\Psi }(d\psi )=\int _{\Psi }\frac{ \pi _{\Psi }(\psi \,\vert \,x)}{\pi _{\Psi }(\psi )}\,\Pi _{\Psi }(d\psi )=\int _{\Psi }\,\Pi _{\Psi }(d\psi \,\vert \,x)=1 \end{aligned}$$

which is a contradiction. (ii) Now suppose $Pl_{\Psi }(x)=\phi ,$ which is true iff $RB_{\Psi }(\psi \,\vert \,x)\le 1$ for every $\psi .$ Since $M(F)=0,$ this implies that for any $x\notin F,$ the set $A(x)=\{\psi :RB_{\Psi }(\psi \, \vert \,x)=1\}$ has $\Pi _{\Psi }(A(x))<1$ which implies $0<1-\Pi _{\Psi }(A(x))=\Pi _{\Psi }(\ Im_\Psi (x)).$ Then

$$\begin{aligned} 1&=\Pi _{\Psi }(A(x))+\Pi _{\Psi }(\ Im_\Psi (x)) \\&>\int _{A(x)}RB_{\Psi }(\psi \,\vert \,x)\,\Pi _{\Psi }(d\psi )+\int _{ \ Im_\Psi (x)}RB_{\Psi }(\psi \,\vert \,x)\,\Pi _{\Psi }(d\psi ) \\&=\int _{\Psi }RB_{\Psi }(\psi \,\vert \,x)\,\Pi _{\Psi }(d\psi )=\int _{\Psi }\,\Pi _{ \Psi }(d\psi \,\vert \,x)=1 \end{aligned}$$

which is a contradiction. $\square$

It is also possible to construct credible regions based on the relative belief ratio as in $C_{\gamma }(x)=\{\psi :RB_{\Psi }(\psi \,\vert \,x)\ge r_{\gamma }\}$ where $r_{\gamma }=\sup \{r:\Pi _{\Psi }(RB_{\Psi }(\psi \,\vert \,x)<r\,\vert \,x)\le 1-\gamma \}$ as then $\Pi _{\Psi }(C_{\gamma }(x)\,\vert \,x)\ge \gamma .$ As with all relative belief inferences, the relative belief credible regions are invariant under smooth reparameterizations while hpd regions are not. This means that the computation of a $\gamma$-relative belief region can be carried out in any parameterization while each parameterization leads to a potentially different hpd credible region. With both approaches, however, it is impossible to say a priori that all the elements of the region will have evidence in their favor. For relative belief regions, however, it is guaranteed that for any $\gamma \le \Pi _{\Psi }(Pl_{\Psi }(x)\,\vert \,x)$ then $C_{\gamma }(x)\subset Pl_{\Psi }(x)$ and there is evidence in favor of each element of $C_{\gamma }(x)$ so such a region can be also be reported. There are also a variety of optimality properties satisfied by relative belief credible regions, see Evans (2015). The property of importance for the discussion here, however, is that for the plausible region it can be determined a priori how much data to collect to ensure appropriate coverage probabilities and that doesn’t seem to be available for a credible region in general.

It is also the case, as established in Evans and Guo (2021), that plausible regions possess additional good, and even optimal, properties beyond those already cited like parameterization invariance and no dependence on the valid measure of evidence used. For example, the prior probability of $Pl_{\Psi }(x)$ covering the true value is always greater than or equal to the prior probability of $Pl_{\Psi }(x)$ covering a false value which in frequentist theory is known as the unbiasedness property for confidence regions. As an example of an optimal property, when the prior $\Pi _{\Psi }$ is continuous, then among all regions *C* satisfying $M(\psi \in C(X)\,\vert \,\psi )\ge M(\psi \in Pl_{\Psi }(X)\,\vert \,\psi )$ for every $\psi ,$ namely, the conditional prior probability that *C* covers the true value is as large as this probability for $Pl_{\Psi },$ then $Pl_{\Psi }$ maximizes the prior probability of not covering a false value and there is a similar optimality property for the discrete case. The implication of this is that, if one considers another way of expressing evidence that leads to the region *C*,  then provided its coverage probabilities are as large as those of $Pl_{\Psi },$ as otherwise it presumably wouldn’t be considered, then *C* cannot do better than $Pl_{\Psi }$ with respect to accuracy. This is really an optimality property for the principle of evidence and there are many other such results.

## Examples

There are a variety of problems discussed in the literature where issues concerning either improper confidence regions are obtained or it is unclear how to construct a $\gamma$-confidence region for a general parameter $\psi =\Psi (\theta ).$ The following examples show that the approach via the principle of evidence can deal successfully with such problems.

### Fieller’s problem

This is a well-known problem, as discussed in Geary (1930), Fieller (1954), Hinkley (1969) and more recently in Pham-Gia et al. (2006) and Ghosh et al. (2006) where a wide range of applications are noted. Ghosh et al. (2006) is concerned with confidence intervals for ratios of regression coefficients in a normal linear model and it is shown that certain integrated likelihoods do not produce improper intervals and this is now a consequence of the general Theorem 1. This problem is also discussed in Fraser et al. (2018) where it appears as problems A and B of a set of problems for inference proposed by D. R. Cox.

For this there are two samples $x=(x_{1},\ldots ,x_{n_{x}})$ *i*.*i*.*d*. $N(\mu ,\sigma _{0}^{2})$ independent of $y=(y_{1},\ldots ,y_{n_{y}})$ *i*.*i*.*d*. $N(\nu ,\sigma _{0}^{2})$ where $(\mu ,\nu )\in {\mathbb {R}} ^{2}$ is unknown. So it is supposed that the means are unknown but the variances are known and common. The discussion can be generalized to allow for unknown variances as well, with no changes to the basic results, but the essential problem arises in the simpler context. The problem then is to make inference about the ratio of means $\psi =\Psi (\mu ,\nu )=\mu /\nu$ and, in particular, construct a confidence interval for this quantity. It is assumed here that model checking has not led to any suspicions concerning the validity of the models. As such the data can be reduced to the minimal sufficient statistic $({\bar{x}},{\bar{y}})$ where ${\bar{x}}\sim N(\mu ,\sigma _{0}^{2}/n_{x})$ independent of ${\bar{y}}\sim N(\nu ,\sigma _{0}^{2}/n_{y}).$

Confidence regions for $\psi$ can be obtained via a pivotal statistic given by $({\bar{x}}-{\bar{y}}\psi )/\sigma _{0}\sqrt{1/n_{x}+\psi ^{2}/n_{y}}\sim N(0,1)$ but this can produce improper regions. For example, if a $\gamma$-confidence interval is required for $\psi$ then, with $z_{p}$ denoting the *p*-th quantile of a *N*(0, 1),  the region equals ${\mathbb {R}} ^{1}$ whenever $\vert {\bar{y}}\vert <\sigma _{0}z_{(1+\gamma )/2}/n_{y}^{1/2}$ and $n_{x}{\bar{x}}^{2}+n_{y}{\bar{y}}^{2}<z_{(1+\gamma )/2}^{2}\sigma _{0}^{2}.$ Sometimes the region can be a so-called *exclusive region* of the form $(-\infty ,a({\bar{x}},{\bar{y}}))\cup (b({\bar{x}},{\bar{y}}),\infty )$ with $a( {\bar{x}},{\bar{y}})<b({\bar{x}},{\bar{y}}).$ While an interval might be preferred, there is nothing illogical about an exclusive region as can be seen by considering the $\gamma$-confidence interval ${\bar{x}}\pm \sigma _{0}z_{(1+ \gamma )/2}/n_{x}^{1/2}$ for $\mu .$ If this interval includes 0,  then necessarily the $\gamma$-confidence region for $1/\mu$ has the exclusive form $(-\infty ,1/({\bar{x}}-\sigma _{0}z_{(1+\gamma )/2}/n_{x}^{1/2}))\cup (1/( {\bar{x}}+\sigma _{0}z_{(1+\gamma )/2}/n_{x}^{1/2}),\infty ).$ The same reasoning applies in Fieller’s problem and one can always reparameterize by making inference instead about $\psi ^{-1}$ to obtain an interval. The problem of exclusive regions is a consequence of the parameterization but that is not the case with improper regions as this represents a defect in the inference.

The relative belief approach requires the specification of a prior and for this conjugate priors $\mu \sim N(\mu _{0},\tau _{10}^{2})$ independent of $\nu \sim N(\nu _{0},\tau _{20}^{2}),$ will be used. This requires an elicitation for the quantities $(\mu _{0},\tau _{10}^{2},\nu _{0},\tau _{20}^{2})$ which can proceed as follows. First specify $(m_{1},m_{2})$ such that the true value of $\mu \in (m_{1},m_{2})$ with virtual certainty, say with prior probability $\gamma =0.99.$  Note that  the data arises via a measurement process and the particular process used is part of the design of the study. For example, the particular instrumentation used places bounds on what the possible measurements will be as these can’t be arbitrarily large. Based on this information $(m_{1},m_{2})$ can be chosen and a small probability is allowed for the true mean to fall outside this interval to reflect the fact that such a specification is not categorical. If this was an inappropriate choice, then this will be detected when a check is made for prior-data conflict (see the following paragraph) and a modification is required. After choosing $(m_{1},m_{2})$ then put $\mu _{0}=(m_{1}+m_{2})/2$ and solve $\Phi ((m_{2}-\mu _{0})/\tau _{10})-\Phi ((m_{1}-\mu _{0})/\tau _{10})= \gamma$ for $\tau _{10},$ which can be done iteratively via bisection, so the prior on $\mu$ is now determined. This step could also be applied to obtain the prior for $\nu$ but it is supposed instead that there is information about the true value of $\psi$ expressed as $\psi \in (r_{1},r_{2})$ with virtual certainty for fixed constants $r_{1}<r_{2}$. A value $\psi _{0}\in (r_{1},r_{2})$ is then selected, which could be a hypothesized value for this quantity or just the central value, and then take $\nu _{0}=\mu _{0}/\psi _{0}.$ Finally, requiring $\nu \in (m_{1}/r_{2},m_{2}/r_{1})$ with virtual certainty determines $\tau _{20}$ via $\Phi ((m_{2}/r_{1}-\nu _{0})/\tau _{20})-\Phi ((m_{1}/r_{2}-\nu _{0})/\tau _{20})=\gamma$ and this gives the prior for $\nu \mathbf {.}$ This is just one method for eliciting the prior and an alternative could be more suitable in a given application.

Once a prior has been determined and the data obtained, the prior is subjected to a check for prior-data conflict and it is assumed here that the prior has passed such a check. For example, as discussed in Evans and Moshonov (2006), a check for the prior on $\mu$ leads to computing the tail probability

5 $$\begin{aligned} M(m({\bar{X}})\le m({\bar{x}}))=2\left( 1-\Phi \left( \vert {\bar{x}}-\mu _{0}\vert / \sqrt{\tau _{10}^{2}+\sigma _{0}^{2}/n_{x}}\right) \right) \end{aligned}$$

since ${\bar{x}}$ is a minimal sufficient statistic with prior distribution ${\bar{x}}\sim N(\mu _{0},\tau _{10}^{2}+\sigma _{0}^{2}/n_{x}).$ If (5) is small, then the observed ${\bar{x}}$ is in the tails of its prior distribution and so indicates a problem with the prior given that the model has passed its checks which are conducted first. It is clear that as $n_{x}\rightarrow \infty ,$ then (5) converges to $2(1-\Phi \left( \vert \mu _{true}-\mu _{0}\vert /\tau _{10}\right)$ which is the tail probability based on the prior for $\mu$ and this measures whether or not the true value of the parameter is in the tails of the prior. A general consistency result for this approach to checking for prior-data conflict is established in Evans and Jang (2011a).

Methodology for replacing a defective prior is developed in Evans and Jang (2011b). This entails specifying, before seeing the data, a hierarchy of progressively more weakly informative priors, starting from a base elicited prior, where the concept of one prior being weakly informative with respect to another is in terms of the new prior producing fewer prior-data conflicts. The degree of weak informativity can be quantified so that a sequence of priors $\pi _{i}$ for $i=0,1,\ldots ,$ can be constructed where $\pi _{0}$ is the base prior, and $\pi _{i}$ is, for example, $20\%$ more weakly informative than $\pi _{i-1}.$ Then, if a prior-data conflict is obtained with $\pi _{0},$ this prior is replaced by one higher up the hierarchy until the conflict is avoided. Strictly speaking these priors are not data dependent but the need to replace a prior is as is the point where this process stops. For the $N(\mu _{0},\tau _{10}^{2})$ prior the hierarchy is given by a sequence of $N(\mu _{0},\tau _{1i}^{2})$ priors where $\tau _{1i}^{2}>\tau _{1i-1}^{2},$ namely, where the priors are increasingly diffuse. The necessary computations for this and other examples can be found in Evans and Jang (2011b).

One concern with checks for the model or prior is whether or not the coverage probabilities for regions need to be adjusted to reflect this step. For the developments here, however, such adjustments are not relevant because the inferences reported are not the coverage probabilities but rather these are the estimate, the plausible region and its posterior content and a measure of its size. The bias computations are measures of the reliability of the inferences reported and indeed, if it is determined that either the model or prior are not appropriate and new ingredients are specified, then these computations need to be performed again based on the new choices and this may entail more data being collected to ensure that the biases are small. It is to be noted too that, even without specifying a prior, the modification of coverage probabilities based on checking the model, a step that is part of good statistical practice, is rarely, if ever, carried out and the approach taken here justifies this as the coverage probabilities are not the inferences reported.

Some numerical examples are carried along for illustration purposes.

#### Example 1

*Simulation example (the data, model and prior).*

Suppose $$n_{x}=n_{y}=10,\mu =20,\nu =10,\sigma _{0}^{2}=1$$ so the true value is $$\psi =20/10=2\mathbf {.}$$  Data was generated leading to the mss $$( {\bar{x}},{\bar{y}})=(20.188,10.699).$$ For the prior elicitation suppose $$ (m_{1},m_{2})=(10,25),$$ so $$\mu _{0}=17.5,\tau _{10}=2.912$$ and with $$ (r_{1},r_{2})=(1,3),\psi _{0}=2\,$$ then $$\nu _{0}=8.75,\tau _{20}=2.336.$$ The value $$\psi _{0}=2$$ is chosen as the hypothesis $$H_{0}:\Psi (\mu ,\nu )=2$$ will be subsequently assessed to see how the approach performs with a true hypothesis. Inverting the pivotal leads to the 0.95- Bayesian confidence region (1.770, 2.016) for $$\psi $$ which just includes the true value. $$\square $$

#### Example 2

*Cox’s examples (the data, model and prior).* For the Cox A problem, $n_{x}=n_{y},\sigma _{0}^{2}/n_{y}=1,({\bar{x}},{\bar{y}} )=(10,0.5)$ which produces the exclusive 0.95-Bayesian confidence region $(-\infty ,-6.752)\cup (3.968,\infty )$ via the pivotal. For Cox B the only change is that now $({\bar{x}},{\bar{y}})=(0.5,0.5)$ and the 0.95-Bayesian confidence region is ${\mathbb {R}} ^{1}$ and so is improper. No priors were prescribed for either problem, so here we take fairly noninformative priors that avoid prior-data conflict. For problem A suppose $\mu \sim N(12,3)$ independent of $\nu \sim N(0,3)$ and for problem B suppose both priors are *N*(0, 3). $\square$

Putting

6 $$\begin{aligned} \tau _{20}^{2}(\psi )=\left( \psi ^{2}/\tau _{10}^{2}+1/\tau _{20}^{2}\right) ^{-1},\nu _{0}(\psi )=\tau _{20}^{2}(\psi )\left( \psi \mu _{0}/\tau _{10}^{2}+\nu _{0}/\tau _{20}^{2}\right) , \end{aligned}$$

then the exact prior density of $\psi$ is

$$\begin{aligned} \pi _{\Psi }(\psi )&=\frac{2\tau _{20}^{2}(\psi )}{\sqrt{2\pi }\tau _{10}\tau _{20}}\exp \left\{ -\frac{1}{2}\frac{\tau _{20}^{2}(\psi )(\mu _{0}-\nu _{0} \psi )^{2}}{\tau _{10}^{2}\tau _{20}^{2}}\right\} \\&\quad \times \left\{ \varphi \left( \frac{\nu _{0}(\psi )}{\tau _{20}(\psi )}\right) +\frac{ \nu _{0}(\psi )}{\tau _{20}(\psi )}\Phi \left( \frac{\nu _{0}(\psi )}{ \tau _{20}(\psi )}\right) -\frac{1}{2}\right\} . \end{aligned}$$

Note that when $\mu _{0}=\nu _{0}=0$ then $\pi _{\Psi }$ is a (rescaled) Cauchy density so in general this distribution has quite long tails. The same formula works for the posterior with substitutions as in (6) since

$$\begin{aligned} \mu \,\vert \,{\bar{x}}&\sim N(\left( \mu ({\bar{x}}),\left( \frac{n_{x}}{ \sigma _{0}^{2}}+\frac{1}{\tau _{10}^{2}}\right) ^{-1}\right) \text { with }\mu ( {\bar{x}})=\left( \frac{n_{x}}{\sigma _{0}^{2}}+\frac{1}{\tau _{10}^{2}}\right) ^{-1}\left( \frac{n_{x}{\bar{x}}}{\sigma _{0}^{2}}+\frac{\mu _{0}}{\tau _{10}^{2}} \right) \\&\text {independent of} \\ \nu \,\vert \,{\bar{y}}&\sim N\left( \nu ({\bar{y}}),\left( \frac{n_{y}}{ \sigma _{0}^{2}}+\frac{1}{\tau _{20}^{2}}\right) ^{-1}\right) \text { with }\nu ( {\bar{y}})=\left( \frac{n_{y}}{\sigma _{0}^{2}}+\frac{1}{\tau _{20}^{2}}\right) ^{-1}\left( \frac{n_{y}{\bar{y}}}{\sigma _{0}^{2}}+\frac{\nu _{0}}{\tau _{20}^{2}} \right) . \end{aligned}$$

So the relative belief ratio $RB_{\Psi }(\psi \,\vert \,{\bar{x}},{\bar{y}})$ is available in closed form.

For a general problem, a closed form is typically not available for the prior and posterior densities of marginal parameters of interest. In an application, however, there is a difference $\delta >0$ that represents the accuracy with which it is desired to know the true value. This quantity is a major input into sample size considerations. The approach then is to partition the effective prior range of $\psi ,$ as determined via a simulation from the prior of $(\mu ,\nu ),$ into subintervals of length $\delta$ with the midpoint of each interval taken as representative of the values in that subinterval. The prior and posterior contents of these subintervals are determined via a simulation and then density histograms are used to approximate $\pi _{\Psi }(\psi )$ and $\pi _{\Psi }(\psi \,\vert \,{\bar{x}},{\bar{y}})$ which in turn gives an approximation to $RB_{\Psi }(\psi \,\vert \,{\bar{x}},{\bar{y}})$ that can be used to determine the inferences.

#### Example 1

*Simulation example (the inferences).*

**Fig. 1:**
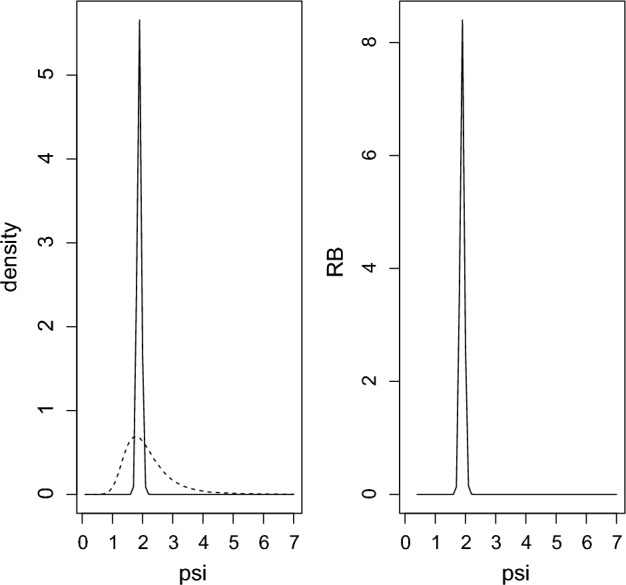
Plots (left panel) of the prior (- - -) and the posterior (–) densities and (right panel) of the relative belief ratio of $\psi$ in Example 1

The above approximation procedure was carried out, using the values recorded when $\delta =0.1$ was chosen for the accuracy. Figure 1 provides plots of $\pi _{\Psi }$ and $\pi _{\Psi }(\cdot \,\vert \,{\bar{x}},{\bar{y}})$ and $RB_{\Psi }(\cdot \,\vert \,{\bar{x}},{\bar{y}}).$ Due to the long-tailed feature of the prior some extreme values of $\psi$ are obtained and this is reflected in the range over which these distribution have been plotted. Relatively smooth estimates are obtained based on Monte Carlo sample sizes of $N=10^{5}$ and these can be seen to closely approximate the true functions. One approach for coping with the long-tail is to calculate the ecdf ${\hat{F}}_{\Psi }$ of $\psi$ based on a large simulation sample and take $(\psi _{\min },\psi _{\max } )=({\hat{F}}_{\Psi }^{-1}(0.0005),{\hat{F}}_{\Psi }^{-1}(0.9995))$ so this ignores 0.001 of the probability in the tails which is what was done here. Another possibility, which avoids the truncation, is to transform to $\omega =G(\psi )$ where *G* is a long-tailed cdf like a Cauchy (or even sub-Cauchy) and transform the initial partition to $(G(\psi _{\min }-\delta /2),G(\psi _{\min }+\delta /2)],\ldots ,(G(\psi _{\max }-\delta /2),G(\psi _{\max }+\delta /2)].$ All inferences for $\psi$ can then be obtained from those for $\omega$ via the transformation $\psi =G^{-1}(\omega )$ due to the invariance of relative belief inferences under reparameterizations.

The relative belief estimate is given by $\psi (x)=1.90$ with plausible region $Pl_{\Psi }(x,y)=(1.75,2.05)$ having posterior content 0.982 and prior content 0.200. So the plausible region contains the true value, and note that the estimate is reasonably accurate for a relatively small amount of data. $\square$

#### Example 2

*Cox’s examples (the inferences).*

For the Cox A problem, the plausible region is $(-\infty ,-17.60)\cup (6.40,\infty )$ having posterior content 0.791 and prior content 0.515. So the inferences are not very precise. For the Cox B problem, the plausible region is $(0.10,\infty )$ having posterior content 0.534 and prior content 0.498 and the improper interval is avoided. Both cases can be considered extreme as there is little data relative to the variance $\sigma _{0}^{2}.$ $\square$

Now consider the bias calculations. To compute the biases for hypothesis assessment it is necessary to compute

7 $$\begin{aligned} M(RB_{\Psi }(\psi _{0}\,\vert \,{\bar{x}},{\bar{y}})\le 1\,\vert \,\psi ) \end{aligned}$$

for various values of $\psi$ where $({\bar{x}},{\bar{y}})$ is generated from the conditional prior predictive given $\psi$ and to compute the biases for estimation we need to be able to compute (7) for values of $\psi _{0}\sim \pi _{\Psi }$ and then average. So it is necessary to: (i) generate $({\bar{x}},{\bar{y}})$ from its conditional prior predictive $M(\cdot \,\vert \,\psi )$ and (ii) compute $RB_{\Psi }(\psi \,\vert \,{\bar{x}},{\bar{y}})$ and compare it to 1.

For (i) the following sequential algorithm will work:

1. generate $\nu \,\vert \,\psi \,\sim \pi (\cdot \,\vert \,\psi ),$ 2. generate ${\bar{y}}\,\vert \,(\psi ,\nu )\sim N(\nu ,\sigma _{0}^{2}/n_{y}),$ 3. generate ${\bar{x}}\,\vert \,(\psi ,\nu ,{\bar{y}})\sim N(\psi \nu ,\sigma _{0} ^{2}/n_{x}).$

Steps 2 and 3 are straightforward while step 1 requires the development of a suitable algorithm and this is done in Appendix B.

To determine (7) the value $RB_{\Psi }(\psi _{0}\,\vert \,{\bar{x}},{\bar{y}})$ needs to be computed for each generated value of $({\bar{x}},{\bar{y}}).$ This can be carried out as previously using the discretized version but using the closed form version is much more efficient. It might seem more appropriate to use the exact form also for inferences but, because we wish to incorporate the meaningful difference $\delta$ into the inferences, the discretized version is much more efficient for those computations. Note too that a high degree of accuracy is not required for the bias computations.

Now consider the biases in the numerical problems being considered.

#### Example 1

*Simulation example (the biases).*

**Fig. 2:**
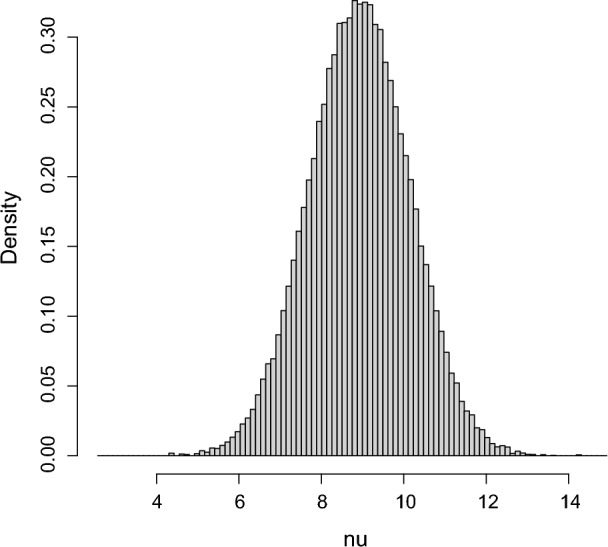
Density histogram of a sample of $10^{5}$ from the conditional prior of $\nu$ given $\psi _{0}=2.$

The hypothesis assessment problem is $H_{0}:\psi _{0}=2$ then, using the elicited values of $(\mu _{0},\tau _{10}^{2},\nu _{0},\tau _{20}^{2}),$ leads to $\tau _{20}^{2}(2)=1.5239,\nu _{0}(2)=8.7502$ and $z_{0}=$ $-7.0883.$ Figure  2 is a density histogram of a sample of $10^{5}$ from $\pi (\cdot \,\vert \,\psi _{0}).$

**Fig. 3:**
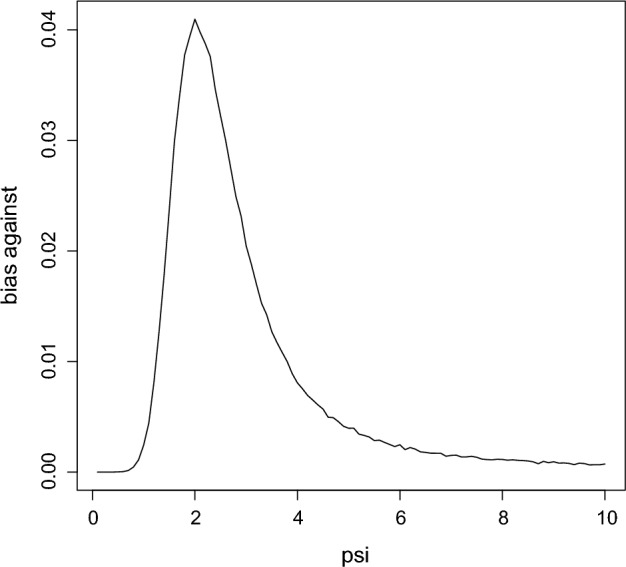
The bias against as a function of $\psi$ in Example 1

To get the bias against $H_{0},$ use the sequential algorithm to generate $({\bar{x}},{\bar{y}})\sim M(\cdot \,\vert \,\psi _{0})$, compute and compare $RB_{\Psi }(\psi _{0}\,\vert \,{\bar{x}},{\bar{y}})$ to 1 for a large number of repetitions recording the proportion of times $RB_{\Psi }(\psi _{0} \,\vert \,{\bar{x}},{\bar{y}})\le 1.$ In this problem the value $M(RB_{\Psi } (\psi _{0}\,\vert \,{\bar{x}},{\bar{y}})\le 1\,\vert \,\psi _{0})=0.04$ was obtained based on a Monte Carlo sample of $10^{5}$ and so there is no real bias against $H_{0}:\psi _{0}=2.$ Figure 3 is a plot of $M(RB_{\Psi } (\psi \,\vert \,{\bar{x}},{\bar{y}})\le 1\,\vert \,\psi )$ versus $\psi$ which is maximized at $\psi =2.0$ and takes the value 0.040 there. This implies that the conditional prior probability the plausible region contains the true value is at least 0.960 for all $\psi$ and so can be considered as a 0.96-confidence interval for $\psi .$ If instead we had $n_{x}=n_{y}=20,$ then the maximum bias against is 0.028 and the plausible region would then be 0.972-Bayesian confidence interval for $\psi$ and of course larger sample sizes will just increase the coverage probability.

**Fig. 4:**
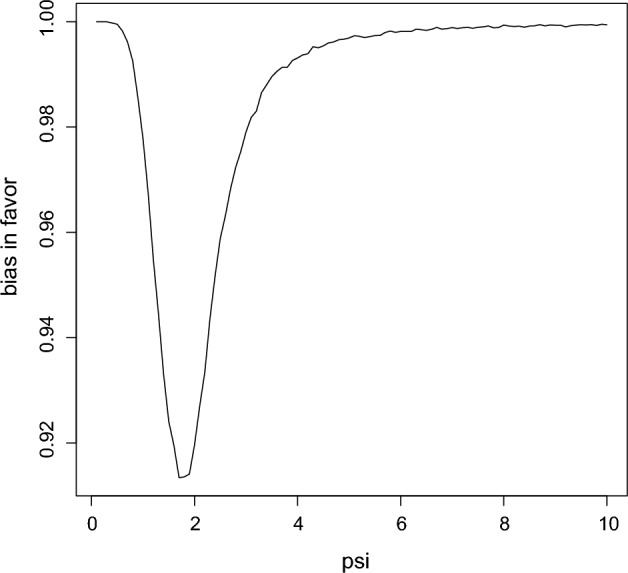
Bias in favor as a function of $\psi$ in Example 1

To get the bias in favor of $H_{0},$ use the sequential algorithm to generate $({\bar{x}},{\bar{y}})\sim M(\cdot \,\vert \,\psi _{0}+\delta /2)$, compute and compare $RB_{\Psi }(\psi _{0}\,\vert \,{\bar{x}},{\bar{y}})$ to 1, for a large number of repetitions, record the proportion of times $RB_{\Psi }(\psi _{0} \,\vert \,{\bar{x}},{\bar{y}})\ge 1,$ and also do this for $({\bar{x}},{\bar{y}})\sim M(\cdot \,\vert \,\psi _{0}-\delta /2)$ and the maximum of the two is an upper bound on the bias in favor. In this case the value $0.92\,$is obtained which is very high indicating that there is substantial bias in favor of the hypothesis. In other words, there is a substantial prior probability that evidence in favor of the hypothesis will be obtained even when it is meaningfully false as determined by $\delta .$ Of course, sample size is playing a role here as well as $\delta .$ For $n_{x}=n_{y}=20$ the upper bound equals 0.91, for $n_{x}=n_{y}=100$ the upper bound equals 0.71, while for $n_{x}=n_{y}=500$ the upper bound equals 0.09. So $n_{x}=n_{y}=10$ is not enough data to ensure that evidence in favor of $H_{0}$ will not be obtained when it is meaningfully false with $\delta =0.1$ and more data needs to be collected to avoid this. For the bias in favor for estimation a sample of $\psi \sim \pi _{\Psi }$ values is generated and the bias in favor of $\psi$ at $\psi \pm \delta /2$ is determined and then averaged. Figure 4 is a plot of the bias in favor as a function of $\psi$ and the average value is 0.94 which is an upper bound on the the prior probability that the plausible region contains a meaningfully false value. When $n_{x}=n_{y}=20$ the upper bound equals 0.92,  when $n_{x}=n_{y}=100$ the upper bound equals 0.69 and when $n_{x}=n_{y}=500$ the upper bound equals 0.26. The value of $\delta$ is determined by the application and taking it too small clearly results in the requirement of overly large sample sizes to get the bias in favor small. For example, with $n_{x}=n_{y}=10$ and $\delta =0.5,$ then the bias in favor for estimation is 0.33 while for $\delta =1.0$ it is 0.12,  and with $n_{x}=n_{y}=20$ these values are 0.21 and 0.07, respectively. $\square$

#### Example 2

*Cox’s examples (the biases).* For the first problem an upper bound on the bias against is given by 0.18 so the coverage probability for the plausible region is at least 0.82. For the second problem an upper bound on the bias against is given by 0.24 so the coverage probability for the plausible region is at least 0.76. These coverages are quite reasonable given the small sample sizes relative to $\sigma _{0}^{2}.$ $\square$

### Mandelkern’s Examples

Mandelkern (2002) discusses several problems in physics where confidence intervals are required but for which no acceptable solution exists. These are problems where standard statistical models are used and, in the unconstrained case, well-known confidence intervals are available for $\psi$ but physical theory demands that the true value lie in $\Psi _{0}\subset \Psi$ for a proper subset $\Psi _{0}.$ Such problems have long been recognized and, for example, Quenouille (1958) discusses inferences for restricted parameters as well as Zhang and Woodroofe (2003) where Bayesian credible intervals based on improper priors are shown to have good coverage probabilities in some contexts.

If *C*(*x*) is a $\gamma$-confidence region for unconstrained $\psi ,$ then it is certainly the case that $C(x)\cap \Psi _{0}$ is a $\gamma$-confidence region under the constraint. While this has the correct coverage probability, however, in general $C(x)\cap \Psi _{0}$ can equal $\phi$ with positive probability and so this solution is improper. As is now demonstrated the approach discussed here provides an effective solution to this problem.

Mandelkern’s examples are now described together with the solutions.

#### Example 3

*Location-normal with constrained mean.*

The model here is that a sample $x=(x_{1},\ldots ,x_{n})$ has been obtained from a distribution in $\{N(\mu ,\sigma _{0}^{2}):\mu \in (l_{0},u_{0})\}$ where $\sigma _{0}^{2}$ is known and $\mu$ is known to lie in the interval $(l_{0},u_{0})$ where $l_{0},u_{0}\in {\mathbb {R}} ^{1}\cup \{-\infty ,\infty \}$ with $l_{0}<u_{0}.$ Mandelkern discusses inferences concerning the mass $\mu$ of a neutrino so in that case $l_{0}=0.$ The measurements are taken to a certain accuracy and this is reflected in the specification of the quantity $\delta$ which is the accuracy to which it is desired to know $\mu$ which may indeed be larger than the accuracy of the measurements. This leads to a grid of possible values for $\mu ,$ say $\mu _{1}<\mu _{2}<\cdots$ and such that $\left| \mu _{i} -\mu _{i+1}\right| =\delta .$ So when $l_{0}$ and $u_{0}$ are both finite the possible values of $\mu$ are given by $\mu _{i}=l_{0}+(i-1/2)\delta$ for $i=1,\ldots ,(u_{0}-l_{0})/\delta ,$ and it is supposed that these values are such that $(u_{0}-l_{0})/\delta$ is an integer. It is certainly possible for one or both of $l_{0},u_{0}$ to be infinite but typically there are lower and upper bounds on what a measurement can equal. So in practice a finite number of such intervals with possibly two tail intervals, which contain very little prior probability, suffices. For example, consider measuring a length to the nearest centimeter so it would make sense to take $\delta =1$ cm and the $\mu _{i}$ are consecutive integer values in centimeters and all values in $[\mu _{i}-\delta /2,\mu _{i}+\delta /2)$ are considered effectively equivalent. For the neutrino problem there is undoubtedly a guaranteed upper bound on the mass. As discussed for Fieller’s problem, priors are chosen via elicitation and continuous priors are considered here with the previously described discretization applied for computations when necessary. Results for two priors are presented for comparison purposes.

The first prior $\pi _{1}$ is taken to be a beta$(\alpha _{0},\beta _{0})$ distribution on the interval $(l_{0},u_{0})$ with the elicitation procedure as described in Evans et al. (2017) although others are possible. For this $\mu =l_{0}+(u_{0}-l_{0})z$ where $z\sim$ beta$(\alpha _{0},\beta _{0}).$ The values of $(\alpha _{0},\beta _{0})$ are specified as follows. First it is required that $\alpha _{0},\beta _{0}\ge 1$ to ensure unimodality and no singularities. Next a proper subinterval $(l_{1},u_{1})\subset (l_{0},u_{0})$ is specified such that $\mu \in (l_{1},u_{1})$ with prior probability $\gamma .$ Typically $\gamma$ will be a large probability (like 0.99 or higher) reflecting the fact that $\mu \in (l_{1},u_{1})$ is known to be true with virtual certainty. Then the mode $m_{0}$ is taken to be equal to a value in $(l_{1},u_{1}),$ such as $m_{0}=(l_{1}+u_{1})/2,$ which implies $m_{0} =l_{0}+(u_{0}-l_{0})(\alpha _{0}-1)/\tau _{0}\in (l,u)$ where $\tau _{0} =\alpha _{0}+\beta _{0}-2.$ This leads to values for $$\alpha _{0}\ $$and $\beta _{0}$ as

$$\begin{aligned} \alpha _{0}=\tau _{0}(m_{0}-l_{0})/(u_{0}-l_{0})+1,\beta _{0}=\tau _{0} (u_{0}-m_{0})/(u_{0}-l_{0})+1 \end{aligned}$$

that are fully specified once $\tau _{0}$ is chosen. The value of $\tau _{0}$ controls the dispersion of the beta$(\alpha _{0},\beta _{0})$ and, with the cdf denoted beta$(\cdot ,\alpha _{0},\beta _{0}),$ we want beta$((u_{1}-l_{0} )/(u_{0}-l_{0}),\alpha _{0},\beta _{0})-$beta$((l_{1}-l_{0})/(u_{0} -l_{0}),\alpha _{0},\beta _{0})=\gamma$, and this is easily solved for $\tau _{0}$ by an iterative procedure based on bisection. For example, with $(l_{0},u_{0})=(0,10),(l_{1},u_{1})=(0.5,9.5),m_{0}=5$ and $\gamma =0.99$ this leads to $(\alpha _{0},\beta _{0})=(2.20,2.20).$ Note that if $(l_{1},u_{1})=(l_{0},u_{0}),$ then use the uniform prior, namely, $\alpha _{0} =\beta _{0}=1$ which is the noninformative case.

The second prior $\pi _{2}$ is taken to be a $N(\mu _{0},\tau _{0}^{2})$ constrained to the interval $(l_{0},u_{0}).$ The interval $(l_{1},u_{1})$ is selected as before and $\mu _{0}\in (l_{1},u_{1})$ is specified while $\tau _{0}$ is chosen to satisfy $(\Phi ((u_{1}-\mu _{0})/\tau _{0})-\Phi ((l_{1}-\mu _{0})/\tau _{0}))/(\Phi ((u_{0}-\mu _{0})/\tau _{0})-\Phi ((l_{0} -\mu _{0})/\tau _{0}))=\gamma$ using the cdf of the prior. As $\tau _{0}\rightarrow 0$ the prior content of $(l_{1},u_{1})$ goes to 1 and as $\tau _{0}\rightarrow \infty$ the limit, using L’Hôpital, is $(u_{1} -l_{1})/(u_{0}-l_{0}).$ So provided $(u_{1}-l_{1})/(u_{0}-l_{0})\le \gamma$ there is a solution for $\tau _{0}.$ For example, with $(l_{0},u_{0} )=(0,10),(l_{1},u_{1})=(0.5,9.5),m_{0}=5$ and $\gamma =0.99$ this leads to $(\mu _{0},\tau _{0}^{2})=(5,1.92^{2}).$

The bias against hypothesis $H_{0}:\mu =\mu _{*}$ is given by $M(RB_{i} (\mu _{*}\,\vert \,{\bar{x}})\le 1\,\vert \,\mu _{*})$ where $M(\cdot \,\vert \,\mu _{*})$ is the $N(\mu _{*},\sigma _{0}^{2}/n)$ measure, $RB_{i}(\mu _{*} \,\vert \,{\bar{x}})=m_{i}({\bar{x}}\,\vert \,\mu _{*})/m_{i}({\bar{x}})$ and $m_{i}(\bar{x})=(n/\sigma _{0}^{2})^{1/2}\int _{l_{0}}^{u_{0}}\varphi \left( n^{1/2} ({\bar{x}}-\mu )/\sigma _{0}\right) \pi _{i}(\mu )\,d\mu$ is the prior predictive density of ${\bar{x}}$ using prior $\pi _{i}.$ The difficulty in evaluating $M(RB_{i}(\mu _{*}\,\vert \,{\bar{x}})\le 1\,\vert \,\mu _{*})$ arises from the need to evaluate $m_{i}({\bar{x}})$ to obtain $RB_{i}(\mu _{*}\,\vert \,{\bar{x}})$ for each ${\bar{x}}$ generated from the $N(\mu _{*},\sigma _{0}^{2}/n)$ distribution. For this we proceed via an approximation where a sample of *N* is obtained from the $M_{i}$ distribution by generating $\mu \sim \pi _{i},{\bar{x}}\,\vert \,\mu \sim N(\mu ,\sigma _{0}^{2}/n),$ the interval $(l_{0} -3\sigma _{0}/\sqrt{n},u_{0}+3\sigma _{0}/\sqrt{n})$ is divided into *k* equal length subintervals and the proportion of ${\bar{x}}$ values falling in each of the *k* intervals is recorded. The probabilities $p_{1},\ldots ,p_{k}$ of these intervals with respect to the $N(\mu _{*},\sigma _{0}^{2}/n)$ are computed and the relative belief ratios $RB(\mu _{*}\,\vert \,{\bar{x}})$ are then estimated by the ratios of the $p_{i}$ to the relevant proportion obtained from sampling from $M_{i}.$ Finally, the $p_{i}$ probabilities for the intervals, where the estimated relative belief ratio is less than or equal to 1, are summed to give the estimate of the bias against. Clearly as *k* and *N* increase this approximation will converge to $M(RB_{i}(\mu _{*}\,\vert \,{\bar{x}})\le 1\,\vert \,\mu _{*}).$ For these computations values of $N=10^{6}$ and of *k* of at least $10^{3},$ where the choice depended on *n*,  were used. For example, with $\sigma _{0}^{2}=1,$ the other constants as previously specified and $\mu _{*}=4,$ Table 1 gives the values of the bias against for different sample sizes and two different priors. It is seen that bias against is not a problem with either prior.

**Table 1:** Bias against values in Example 3 for testing $H_0:\mu _*=4$ for various sample sizes

*n*	Bias against $H_{0}:\mu _{*}=4$ with $\pi _{1}$	Bias against $H_{0}:\mu _{*}=4$ with $\pi _{2}$
10	0.039	0.047
20	0.026	0.032
50	0.015	0.019
100	0.011	0.013
500	0.004	0.005

**Fig. 5:**
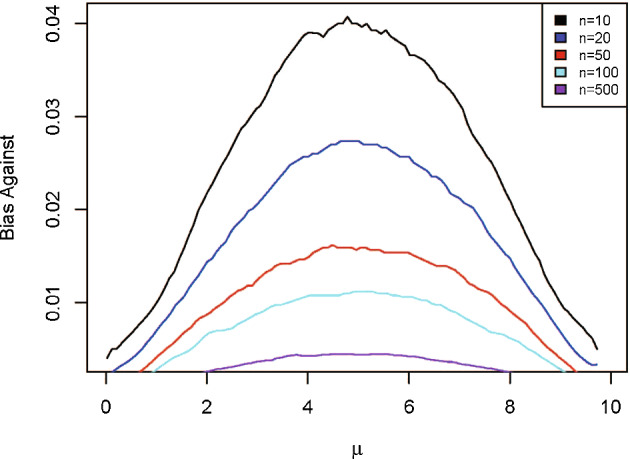
Bias against in Example 3 when using prior $\pi _{1}$ for various sample sizes

**Fig. 6:**
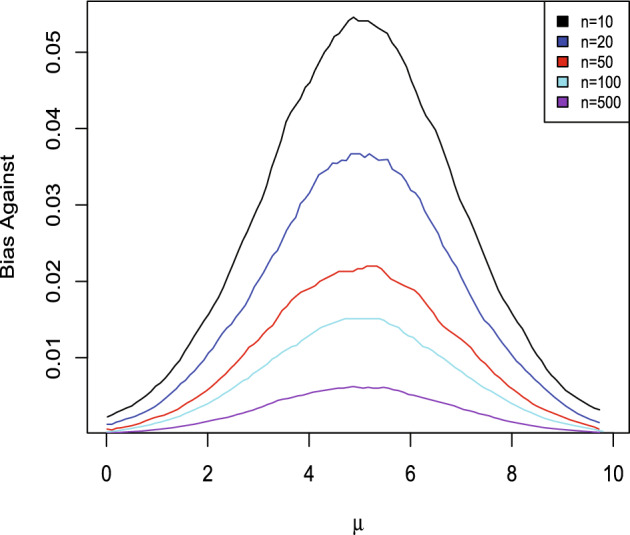
Bias against in Example 3 when using prior $\pi _{2}$ for various sample sizes

Figure 5 is a graph of $M(RB_{i}(\mu \,\vert \,{\bar{x}})\le 1\,\vert \,\mu )$ as a function of $\mu$ when using $\pi _{1}$ for various *n* and Fig. 6 is the graph using $\pi _{2}.$ It is seen that the bias against is maximized at a value $\mu _{\max }$ which implies that $M(RB_{i} (\mu _{\max }\,\vert \,{\bar{x}})\le 1\,\vert \,\mu _{\max })$ serves as an upper bound on the bias against for estimation purposes. As such $M(RB_{i}(\mu _{\max }\,\vert \,\bar{x})>1\,\vert \,\mu _{\max })$ is a lower bound on the coverage probabilities $M(\mu \in Pl_{i}({\bar{x}})\,\vert \,\mu )$ for $$\mu \in (l_{0},u_{0})\ $$where $Pl_{i}({\bar{x}})$ is the plausible region based on $\pi _{i}.$ Therefore $Pl_{i}({\bar{x}})$, as a Bayesian and frequentist confidence region, has coverage probability $M(RB_{i}(\mu _{\max }\,\vert \,{\bar{x}})>1\,\vert \,\mu _{\max })$ or greater. Table 2 contains the confidence values for $Pl_{i}({\bar{x}})$ for various sample sizes. So it is seen that a $95\%$ frequentist coverage is achieved fairly easily. It is to be noted, however, that the confidence is a priori and the correct measure of belief that the true value is in $Pl_{i}({\bar{x}}),$ based on the principle of conditional probability, is the posterior probability. The Bayesian a priori coverage probability for $Pl_{i}({\bar{x}})$ is also recorded in Table 2. In many ways these coverage probabilities can be considered as more appropriate than the pure frequentist coverage as they take into account what is known about $\mu$ through the prior. There is very little difference in this example.

**Table 2:** Frequentist and Bayesian confidence that $Pl_i({\bar{x}})$ contains the true value in Example 3 for various sample sizes

*n*	$$\begin{array}{c}\text {Confidence level of }Pl({\bar{x}})\\ \text {using }\pi _{1}\text { (Bayes)} \end{array} $$	$$ \begin{array}{c}\text {Confidence level of }Pl({\bar{x}})\\ \text {using }\pi _{2}\text { (Bayes)} \end{array}$$
10	$0.958 \,\,\,(0.969)$	$0.945\,\,\,(0.961)$
20	$0.973\,\,\,(0.979)$	$0.962\,\,\,(0.974)$
50	$0.984\,\,\,(0.988)$	0.977 (0.985)
100	$0.988\,\,\,(0.991)$	$0.985\,\,\,(0.989)$
500	$0.995\,\,\,(0.997)$	$0.994\,\,\,(0.996)$

**Fig. 7:**
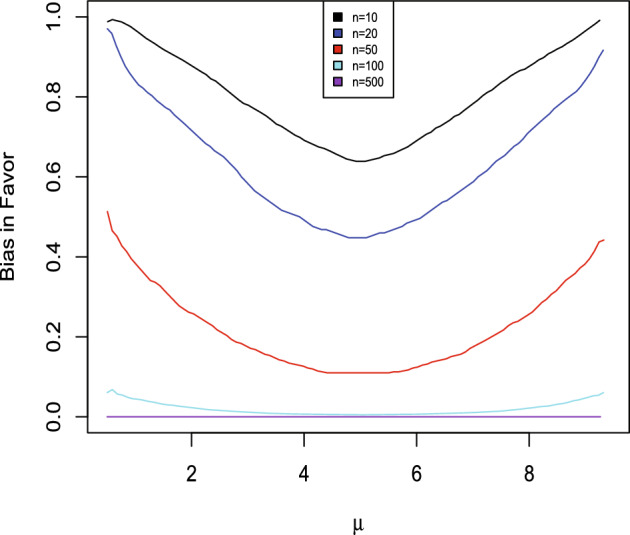
Bias in favor in Example 3 with prior $\pi _{2}$ for various *n* and $\delta .$

It is also necessary to be concerned about bias in favor of $H_{0}:\mu =\mu _{*}.$ Generally this bias is the more serious concern because of a predilection towards the use of diffuse priors as these generally induce bias in favor. Table 3 presents the bias in favor for different *n* and $\delta$ and Fig. 7 is a plot of the bias in favor when using $\pi _{2}$ and a similar plot is obtained with $\pi _{1}.$ Similar results are obtained for the bias in favor for estimation as presented in Table 4. It is seen that the bias in favor in both problems can be substantial and for a given prior and $\delta$ this can only be decreased by increasing the sample size. One needs to be realistic about what accuracy is necessary, both to make sure the study is returning results of sufficient accuracy and that resources are not being wasted. For estimation the bias in favor is measured by averaging the bias in favor of a false value with respect to the prior. As Table 4 indicates, large sample sizes are needed to make sure the bias in favor is small, although this can be mitigated by taking $\delta$ larger. $\square$

**Table 3:** Bias in favor of $\mu _*$ in Example 3 for various sample sizes and meaningful differences

*n*	$$\begin{array}{c} \text {Bias in favor of }H_{0}:\mu _{*}=4\\ \text {using }\pi _{1},\,\delta =0.5\,(0.1) \end{array} $$	$$\begin{array}{c} \text {Bias in favor of }H_{0}:\mu _{*}=4\\ \text {using }\pi _{2},\,\delta =0.5\,(0.1) \end{array}$$
10	$0.700 \,\,\, (0.954)$	0.686 (0.947)
20	$0.515 \,\,\, (0.962)$	0.495 (0.957)
50	$0.136 \,\,\, (0.957)$	0.123 (0.951)
100	$0.008\,\,\, (0.941)$	0.006 (0.934)
500	$0.000\,\,\, (0.744)$	0.000 (0.716)

**Table 4:** Bias in favor for estimation in Example 3 for various sample sizes and meaningful differences

*n*	$$ \begin{array}{c} \text {Bias in favor for estimation}\\ \text {using }\pi _{1},\,\delta =0.5\,(0.1) \end{array} $$	$$ \begin{array}{c} \text {Bias in favor for estimation}\\ \text {using }\pi _{2},\,\delta =0.5\,(0.1) \end{array}$$
10	$0.756\,\,\,(0.965)$	0.686 (0.947)
20	$0.569\,\,\,(0.969)$	0.495 (0.956)
50	$0.176\,\,\,(0.966)$	0.123 (0.951)
100	$0.012\,\,\,(0.951)$	0.006 (0.934)
500	$0.000\,\,\,(0.763)$	0.000 (0.716)

#### Example 4

*Poisson with constrained mean.*

Suppose that count measurements $x_{1},\ldots ,x_{n}$ are *i*.*i*.*d*. Poisson$(\lambda )$ where $\lambda$ is known to lie in the interval $(l_{0},u_{0}).$ The Poisson distribution arises as follows: suppose an event occurs in a time interval of length 1 unit with probability *p* and there are *N* independent opportunities for such events to occur. Then $\lambda \approx Np$ for large *N* and so $\lambda$ represents the rate at which the event occurs in such a time interval. As discussed in Mandelkern (2002) sometimes this rate is known to be at least $l_{0}>0$ and, as with the normal example, without loss of generality, it will be supposed that $u_{0}$ is finite as well. Again it is necessary to specify $\delta >0$ such that two values of $\lambda$ that differ less than this are effectively equivalent and also specify the grid of $\lambda _{i}$ values as was done as in Example 3.

Many possibilities exist for a prior $\pi$ but attention is restricted here to a gamma$_{rate}(\alpha _{0},\beta _{0})$ prior. Again an interval $(l_{1},u_{1})\subset (l_{0},u_{0})$ is specified together with a probability $\gamma =\Pi ((l_{1},u_{1}))$ and the mode $m_{0}\in (l_{1},u_{1}).$ Any value in $(l_{1},u_{1})$ is allowed for the mode but the value $m_{0}=(l_{1}+u_{1})/2$ is selected here. Then $m_{0}=(\alpha _{0}-1)/\beta _{0}$ and $\pi$ is a gamma$_{rate}(1+m_{0}\beta _{0},\beta _{0})$ with $\beta _{0}$ determined by $\gamma$ which can be solved for iteratively using bisection. As a specific example suppose $(l_{0},u_{0})=(3,10)$ and $(l_{1},u_{1})=(3.5,9.5).$ This implies that the prior is a gamma$_{rate}(37.20,5.57)$ distribution.

The bias against $H_{0}:\lambda =6.2$ is recorded in Table 5 and for modest sample sizes it is seen that this is well-controlled. Table 6 provides the lower bounds on the Bayesian and frequentist confidence levels and the exact Bayesian coverages for the plausible interval for various *n* and $\delta .$ The coverage probabilities are reasonable for $n\ge 10.$

**Table 5:** Bias against values in Example 4 for testing $H_0:\lambda =6.2$ for various sample sizes and meaningful differences

*n*	Bias against $H_{0}:\lambda =6.5$ with $\delta =0.5(1)$
1	$0.287\,(0.286)$
10	$0.193\,(0.180)$
20	$0.085\,(0.045)$
50	$0.045\,(0.011)$
100	$0.001\,(0.000)$
500	$0.000\,(0.000)$

**Table 6:** Frequentist and Bayesian confidence that $Pl_i({\bar{x}})$ contains the true value in Example 4 for various sample sizes and meaningful differences

*n*	$$ \begin{array}{c}\text {Confidence level of }Pl({\bar{x}})\\ \text {using }\pi ,\delta =0.5\text { (Bayes)} \end{array} $$	$$\begin{array}{c} \text {Confidence level of }Pl({\bar{x}})\\ \text {using }\pi ,\delta =1.0\text { (Bayes)} \end{array}$$
1	$0.581\,(0.667)$	$0.614\,(0.663)$
10	$0.811 \,\,\, (0.840)$	0.828 (0.858)
20	$0.843 \,\,\, (0.878)$	0.865 (0.903)
50	$0.908 \,\,\, (0.935)$	0.948 (0.966)
100	$0.950 \,\,\, (0.966)$	0.986 (0.991)
500	$0.998 \,\,\, (0.999)$	0.998 (1.000)

Table 7 presents values of the bias in favor of the hypothesis $H_{0}:\lambda =6.2$ for various *n* and $\delta .$ It is seen that there is appreciable bias in favor of $H_{0}$ when $\delta =0.5$ unless $n\ge 500.$ When $\delta =1.0,$ however, much smaller sample sizes give reasonable values. Table 8 provides values of for the bias in favor for estimation for various *n* and $\delta$ and large sample sizes are needed to get the bias in favor down to acceptable levels. $\square$

**Table 7:** Bias in favor of $H_0: \lambda =6.2$ in Example 4 for various sample sizes and meaningful differences

*n*	$\text {Bias in favor of }H_{0}:\lambda =6.2$ $\text {using }\pi ,\delta =0.5\,(1.0)$
1	$0.781\ (0.840)$
10	0.800 (0.673)
20	0.667 (0.218)
50	$0.522\,(0.074)$
100	0.105 (0.000)
500	0.016 (0.000)

**Table 8:** Bias in favor for estimation in Example 4 for various sample sizes and meaningful differences

*n*	Bias in favor for estimation using $\pi ,\delta =0.5\,(1.0)$
1	$0.732\,(0.744)$
10	0.865 (0.778)
20	0.850 (0.652)
50	0.775 (0.404)
100	0.662 (0.197)
500	0.204 (0.002)

## Conclusions

The approach taken here to the construction of regions, whether confidence or credible, is somewhat different than what is typically done where a probability $\gamma$ is stated, whether as a coverage probability or as a posterior probability, and then the region is constructed based on the observed data and this probability. Rather, using the principle of evidence, the plausible region is obtained as consisting of those values for which there is evidence in favor of these being the true value and then quoting the posterior probability of the region as a measure of the degree of belief that the true value is in the stated region. Confidence here is an a priori concept which the experimenter uses, before the data is collected, to ensure the experiment will lead to reliable results.

Mandelkern (2002) states five desiderata that an assessment of the accuracy of an estimate via a confidence interval should satisfy. These are now stated with an assessment of how well the methodology described here meets a requirement.

(i) *Confidence bounds are determined using a well-defined principle, which is neither arbitrary nor subjective. *The bounds stated here are fully determined by the principle of evidence. This principle is universal in the sense that it is applicable to all statistical problems and is not tailored to problems with bounded parameters. No optimality criteria are required although the regions obtained do have optimal properties.

(ii) *They do not depend upon prior knowledge of the parameter apart from its domain. *The principle of evidence requires that a proper prior probability distribution be stated so the methodology presented here does not satisfy this. It is to be noted, however, that the methodology used here includes an elicitation algorithm for the choice of the prior, the measurement and control of the bias induced by the prior-model combination and the checking of the model and prior against the data to see if they are contradicted. It is also the case that the check on the prior is a check on any bounds assumed for the parameter. Objectivity is a necessary aspect of scientific work although difficult to characterize precisely. For example, frequentist methods are not objective as these involve subjective choices made by a statistician. While objectivity is the ideal it is necessary to recognize that, while it is unattainable, it can be approached via methodologies that check subjective choices against the objective data and measure and control the bias that such choices may induce.

(iii) *They are equivariant under one-to-one transformation of the data. *The inference methods described here are fully invariant under *all* smooth reparameterizations. The intervals are (integrated) likelihood intervals but with the additional characteristic that these intervals contain only values for which there is evidence in favor of being the true value. Confidence, likelihood and credible intervals do not possess this property.

(iv) *They convey an estimate of the experimental uncertainty. *In addition to their lengths, the intervals here satisfy a Bayesian confidence condition as well as providing the posterior probability that the true value is in the interval. The Bayesian confidence is seen as an a priori assessment of the quality of the experiment while the posterior probability and the length of the interval are assessments of the accuracy of the estimate based upon the observed data. No matter what data is obtained, these intervals are never improper.

(v) *They correspond to a precise statement of probability. *The Bayesian a priori coverage is precise and a precise (sharp) lower bound is determined for the conditional, given the true value, prior coverage. These coverages can be set a priori by choice of sample size. The intervals also have a precise posterior probability which is the correct measure of belief that the interval based on the observed data contains the true value. The biases are a priori probabilities that provide an assessment of the quality of an experiment. So, for example, if the a priori coverage is low, then this suggests that the results have to be treated with caution even if the interval is short and has a high posterior probability.

## Appendix A: Algorithms for the coverages

The following general computational procedure can be used to determine the coverage probabilities of $Pl_{\Psi }(x)=\{\psi :RB_{\Psi }(\psi \,\vert \,x)>1\}$ as given by (3) and (4). 1. Based on a sample from $\Pi _{\Psi }$ construct a grid $\psi _{1}<\psi _{2}<\cdots <\psi _{m}$ that effectively spans the support of $\Pi _{\Psi }$ and is such that $d(\psi _{i},\psi _{i+1})=\delta$ and then estimate the prior contents of the intervals $(\psi _{1}-\delta /2,\psi _{1}+\delta /2],\ldots ,(\psi _{m}-\delta /2,\psi _{m}+\delta /2].$ 2. For each $\psi _{i}$ generate $x_{i}\sim M(\cdot \, \vert \,\psi _{i}),$ which can be done by generating $\theta \sim \Pi (\cdot \, \vert \,\psi _{i}),x_{i}\sim f_{\theta }.$ Then generate a sample from the posterior $\Pi _{\Psi }(\cdot \, \vert \,x_{i})$ and estimate the posterior contents of $(\psi _{1}-\delta /2,\psi _{1}+\delta /2],\ldots ,(\psi _{m}-\delta /2,\psi _{m}+\delta /2].$ 3. Use the estimates obtained in steps 1 and 2 to construct estimates of $RB_{\Psi }(\psi _{j}\,\vert \,x_{i})$ for $j=1,\ldots ,m.$ 4. Repeat steps 2 and 3 many times to approximate $M(RB_{\Psi }(\psi _{i}\, \vert \,x)\le 1\, \vert \,\psi _{i})$ and $\max _{j:d(\psi _{i},\psi _{j})\ge \delta }\{M(RB_{\Psi }(\psi _{i}\, \vert \,x)\ge 1\, \vert \,\psi _{j})\}$ for $i=1,\ldots ,m.$ 5. Compute the averages

$$\begin{aligned}&\sum _{i=1}^{m}M(RB_{\Psi }(\psi _{i}\, \vert \,x)>1\, \vert \,\psi _{i})\pi _{\Psi }(\psi _{i})\delta , \\&\sum _{i=1}^{m}\max _{j:d(\psi _{i},\psi _{j})\ge \delta }\{M(RB_{\Psi }(\psi _{i}\, \vert \,x)\ge 1\, \vert \,\psi _{j})\}\pi _{\Psi }(\psi _{i})\delta \end{aligned}$$

to estimate (3) and (4) respectively where the estimates of the quantities in the sums are obtained in steps 1–4.

As described in the text, in step 4 estimating $\max _{i}M(RB_{\Psi }(\psi _{i}\, \vert \,x)\le 1\, \vert \,\psi _{i})$ gives a lower bound on the coverage probability of $Pl_{\Psi }(x)$ with respect to the model $\{M(\cdot \, \vert \,\psi ):\psi \in \Psi \}$ and this is a pure frequentist coverage when $\Psi (\theta )=\theta .$ This algorithm can be made more efficient when there are closed form expressions available that can be used instead of approximations. Also, the bias calculations do not have to be highly accurate as rough estimates clearly illustrate whether or not there is substantial bias.

## Appendix B: Algorithm for Fieller’s problem

The joint prior density of $(\psi ,\nu ,{\bar{x}},{\bar{y}})$ is proportional to

$$\begin{aligned} \vert \nu \vert \exp \left\{ -\frac{1}{2}\left[ \frac{m({\bar{x}}-\psi \nu )^{2}+n(\bar{y}-\nu )^{2}}{\sigma _{0}^{2}}+\frac{(\psi \nu -\mu _{0})^{2}}{\tau _{10}^{2} }+\frac{(\nu -\nu _{0})^{2}}{\tau _{20}^{2}}\right] \right\} \end{aligned}$$

which implies that $\pi (\nu \,\vert \,\psi )\propto \vert \nu \vert \exp \{ -( \nu -\nu _{0}(\psi )) ^{2}/2\tau _{20}^{2}(\psi )\}$ where $\tau _{20}^{2}(\psi )$ and $\nu _{0}(\psi )$ are as specified in (6). Therefore, $\pi (\cdot \,\vert \,\psi )$ is close to a normal density but for the factor $\vert \nu \vert .$ Transforming $\nu \rightarrow z=\left( \nu -\nu _{0}(\psi )\right) /\tau _{20}(\psi )$ we need to be able to generate *z* from a density of the form $g(z)\propto \vert \nu _{0}(\psi )+\tau _{20}(\psi )z\vert \varphi (z)\propto \vert z-z_{0}\vert \varphi (z)$ where $z_{0}=-\nu _{0}(\psi )/\tau _{20}(\psi )$. Using $d\varphi (z)/dz=-z\varphi (z),$ then $\int _{-\infty }^{z_{0} }-(z-z_{0})\varphi (z)\,dz=z_{0}\Phi (z_{0})+\varphi (z_{0})$ and $\int _{z_{0} }^{\infty }(z-z_{0})\varphi (z)\,dz=-z_{0}(1-\Phi (z_{0}))+\varphi (z_{0})$ which implies

$$\begin{aligned} g(z)&=p(z_{0})I_{(-\infty ,z_{0}]}(z)g_{1}(z)+(1-p(z_{0}))I_{(z_{0} ,\infty )}(z)g_{0}(z),\text { where}\\ g_{1}(z)&=\frac{(z_{0}-z)\varphi (z)}{z_{0}\Phi (z_{0})+\varphi (z_{0} )}\text { when }z\le z_{0},\\ g_{0}(z)&=\frac{(z-z_{0})\varphi (z)}{-z_{0}(1-\Phi (z_{0}))+\varphi (z_{0} )}\text { when }z>z_{0},\text { and }\\ p(z_{0})&=\frac{z_{0}\Phi (z_{0})+\varphi (z_{0})}{-z_{0}+2(z_{0}\Phi (z_{0})+\varphi (z_{0}))}. \end{aligned}$$

So with probability $p(z_{0}),$ generate *z* from $g_{1}$ and otherwise generate *z* from $g_{0}\mathbf {.}$ The cdf of $g_{1}$ for $z\le z_{0}$ equals

$$\begin{aligned} G_{1}(z)=\int _{-\infty }^{z}g_{1}(x)\,dx=\frac{z_{0}\Phi (z)+\varphi (z)}{z_{0}\Phi (z_{0})+\varphi (z_{0})} \end{aligned}$$

and to generate from $g_{1}$ via inversion generate $u\sim U(0,1)$ and solve $G_{1}(z)=u$ for *z* by bisection. To start the bisection set $z_{up}=z_{0}$ and for some $\epsilon >0$ iteratively evaluate $G_{1}(-i\vert z_{0}-\epsilon \vert )$ for $i=0,1,\ldots$ until $G_{1}(-i\vert z_{0}-\epsilon \vert )\le u$ setting $z_{low} =-i\vert z_{0}-\epsilon \vert$ as this guarantees $G_{1}(z_{low})\le u\le G_{1} (z_{up})=1$ so bisection will work. The cdf of $g_{0}$ is for $z>z_{0}$

$$\begin{aligned} G_{0}(z)=\int _{z_{0}}^{z}g_{0}(x)\,dx=\frac{z_{0}(\Phi (z)-\Phi (z_{0} ))+(\varphi (z)-\varphi (z_{0}))}{z_{0}(1-\Phi (z_{0}))-\varphi (z_{0})} \end{aligned}$$

and for this start bisection with $z_{low}=z_{0}$ and iteratively evaluate $G_{0}(i\vert z_{0}+\epsilon \vert )$ until $u\le G_{0}(i\vert z_{0}+\epsilon \vert )$ setting $z_{up}=i\vert z_{0}+\epsilon \vert$ so bisection will work. Finally, when *z* is obtained put $\nu =\nu (\psi )+\tau _{20}(\psi )z$ to get the appropriately generated value of $\nu .$ An interesting consequence of this algorithm is that it must be true that $0<-z(1-\Phi (z))+\varphi (z)$ for every *z* and this implies the well-known *Mills ratio inequality* $(1-\Phi (z))/\varphi (z)<1/z$ when $z\ge 0$ and $\Phi (z)/\varphi (z)<1/\vert z\vert$ when $z\le 0$ which gives useful bounds on tail probabilities for the normal distribution when $\vert z\vert$ is large.
